# Morphological investigations of posttraumatic regeneration in *Timarete* cf. *punctata* (Annelida: Cirratulidae)

**DOI:** 10.1186/s40851-015-0023-2

**Published:** 2015-08-06

**Authors:** Michael Weidhase, Conrad Helm, Christoph Bleidorn

**Affiliations:** Molecular Evolution & Animal Systematics, Institute of Biology, University of Leipzig, Talstraße 33, D-04103 Leipzig, Germany; Sars International Centre for Marine Molecular Biology, Thormøhlensgt. 55, N-5008 Bergen, Norway

**Keywords:** cLSM, Musculature, Nervous system, Polychaetes, Sedentaria

## Abstract

**Introduction:**

Annelids exhibit great regenerative abilities, which are mainly used after injury or during reproduction. These lophotrochozoans thus represent excellent models for regeneration research. However, detailed morphological studies concerning annelid musculature and nervous system redevelopment are limited to few taxa, and do not allow for broader comparisons and general conclusions regarding common patterns amongst annelids.

**Results:**

Using immunohistochemical staining combined with confocal laser scanning microscopy (cLSM), we investigated the redevelopment of body wall musculature and nervous system during anterior and posterior posttraumatic regeneration in *Timarete* cf. *punctata*. Both regeneration processes start with wound healing, blastema formation, and blastema patterning. In posterior regeneration, this leads to the development of a new pygidium and a segment addition zone (SAZ) anterior to this structure. New segments are subsequently added in a sequential fashion. Anterior regeneration in contrast shows the formation of a new prostomium and peristomium first, followed by the simultaneous redevelopment of three segments, and an additional three segments in sequential order. Anterior muscular regeneration shows an outgrowth of longitudinal musculature from the residual body wall musculature, while circular musculature develops independently within the blastema. During posterior regeneration, new musculature becomes visible when the new segments reached a certain age. Neuronal regeneration begins with neurite outgrowth from the old ventral nerve cord in both cases, which are later forming loop structures. In anterior regeneration, the brain redevelops at the anteriormost position of the loops.

**Conclusions:**

Posterior regeneration recapitulates normal growth from a certain timepoint with serial segment development by a posterior segment addition zone. Anterior regeneration is more complex, showing similarities to larval development in matters of the order, in which prostomium, peristomium, and segments are generated. Furthermore, we demonstrate the usefulness of regeneration studies to investigate morphological structures and evolutionary processes.

**Electronic supplementary material:**

The online version of this article (doi:10.1186/s40851-015-0023-2) contains supplementary material, which is available to authorized users.

## Introduction

Annelids exhibit remarkable capacity for regeneration [1, 2]. Although this ability is limited or absent in some taxa, anterior and posterior regeneration is widespread in annelids and was presumably present in their last common ancestor [1, 3, 4]. Annelids can renew structures, as for example seen in the compensatory regeneration of the tube sealing operculum in *Hydroides* species (Serpulidae) [5, 6] or the replacement of chaetae [7, 8]. Posttraumatic regeneration, the replacement of lost body structures after injury, is found to be widely distributed across the annelid tree as well [1, 4]. Regenerative processes are also observed during asxeual and sexual reproduction. Most annelid taxa showing asexual reproduction exhibit high regenerative ability [3]. Asexual reproduction is widespread in annelids and comprises processes such as architomic and paratomic fission [9]. Examples of sexual reproductive modes coupled with regenerative ability, known as schizogamy, can be found in only a few taxa, such as Eunicidae and Syllidae [9, 10].

In Cirratulidae, many members are known to reproduce by architomy, the separation of body fragments prior to the regeneration of anterior and/or posterior body end [11–13]. This mode is also found in the genus *Timarete*, as known for *T. punctata*, a widely distributed cirratulid, which is thought to represent a complex of closely related species [12, 14–17]. In this study, we describe the process of anterior and posterior posttraumatic regeneration in a member of the *T. punctata* complex. We investigated the redevelopment of musculature and nervous system by using immunohistochemical staining techniques combined with subsequent confocal laser scanning microscopy (cLSM). We further describe and discuss the adult myo- and neuroanatomy in comparison with those in other taxa, and report external morphological changes during regeneration. Finally, based on these new observations, we highlight general patterns of regeneration in annelids.

## Materials and methods

### Origin of investigated specimens and workflow of regeneration experiments

**Fig. 1:**
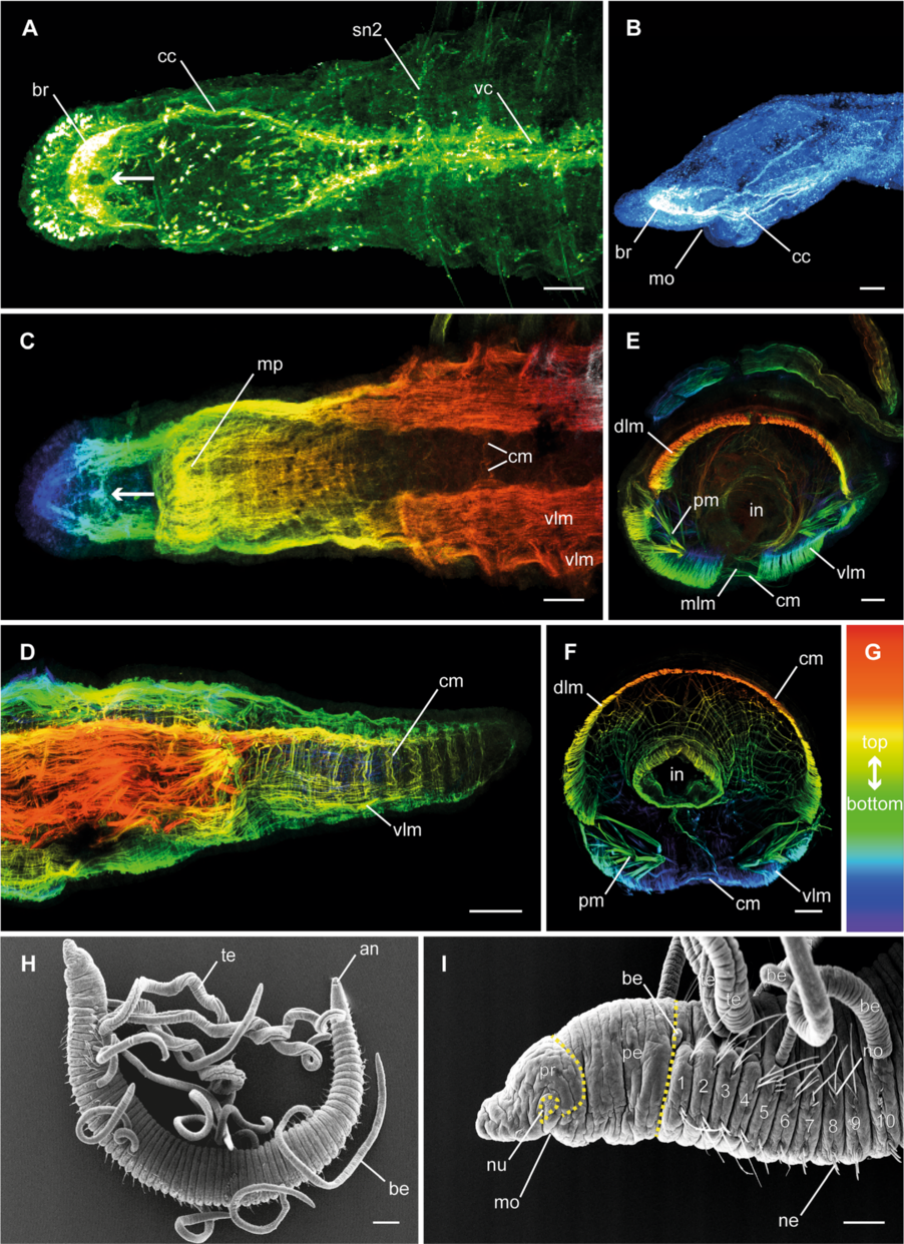
Adult specimens of *Timarete* cf. *punctata*. Anti-FMRFamide (green; A), anti-serotonin (blue; B), and anti-f-actin (depth coded, legend in G; C-F) staining, confocal maximum projections, as well as SEM overviews (H-I). Anterior is left (A-D, I) respectively upper left (H). **a** Anterior end in ventral view, showing the brain (br), circumesophageal connectives (cc), the ventral nerve cord (vc), and the segmental nerve 2 (sn2) in each segment. The arrow indicates the circular area without FMRFamide-immunoreactivity. **b** Lateral view of the pro- and peristomium visualizing the orientation of the brain (br). **c** Anterior ventral view, showing ventral longitudinal musculature (vlm), circular musculature (cm), and the muscular pouch of the mouth opening (mp). The arrow indicates the dorso-ventral muscle fibers penetrating the brain. **d** Posterior end in ventro-lateral view, showing the ventral longitudinal (vlm) as well as circular musculature (cm). **e**, **f** Cross-sections of anterior (E) and posterior (F) segments showing dorsal (dlm) and ventral (vlm) longitudinal musculature, circular musculature (cm), the median ventral longitudinal muscle fiber (mlm) and the musculature associated with the parapodia (pm). Also meshwork-like musculature surrounding the intestine (in) is visible. **h**, **i** Overview of external anatomy (H) and detail of the anterior end (I), showing location and shape of prostomium (pr), peristomium (pe), the first ten chaetigers (1–10), mouth opening (mo), nuchal organ (nu), noto- (no) and neurochaetae (ne), as well as branchiae (br) and tentacles (te). Scale bars = 100 μm, except H = 200 μm

Specimens identified as *Timarete punctata* (Fig. 1h, i) were discovered in a seawater aquarium at the University of Leipzig. As, according to Magalhães et al. [17] *T. punctata* (Grube, 1859) represents a species complex, we used a DNA barcoding approach to characterize our specimens by sequencing fragments of the CO1 and 16S (see [18] concerning barcoding approach). Sequences for the specimens used, a maximum likelihood based phylogenetic reconstruction using the GTR + Γ + I model of sequence evolution, as well as pairwise sequence distances are given in the Additional file 1: Supplementary Information and Additional file 2: Figure S1.

Prior to the experiments, specimens were separated in a 30 l aquarium containing artificial sea water at a temperature of 27 ± 1 °C, sandy sediment, and a circulation pump. Within less than one month, they showed an enormous increase in number through asexual reproduction by architomy (it should be noted, that one year before the experiments also specimens with eggs were observed).

For performing regeneration experiments, specimens having a length between 10 and 15 mm were anesthetized in a solution containing 3.5 % MgCl_2_ in artificial sea water for about 10 min. Afterwards they were dissected between the tenth and eleventh chaetiger using a hollow needle. The anterior and posterior body parts were separated in PS boxes (Rotilabo®-Frischhaltebox Gerda, 1000 ml, Carl Roth GmbH, Karlsruhe, Germany) containing artificial seawater at a temperature of 27 ± 1 °C and an air influx. To enable daily fixation and to avoid inhomogeneity, based for example on long retention time in MgCl_2_ solution, this was done by two sets of experiments (Table 1, set 1 and 2). For fixation, specimens were anesthetized in 7 % MgCl_2_ dissolved in artificial sea water and subsequently fixed in a solution of 4 % paraformaldehyde in 0.1 M PBS (phosphate buffer solution, pH 7.4) overnight at 4 °C. After several rinses in 0.1 M PBS for at least 3 h at RT (room temperature), specimens were stored in PBS-azide (0.1 M PBS containing 0.02 % NaN_3_) at 4 °C until usage. Because first cLSM analyses revealed that fixation every day was not enough to illuminate all aspects of nervous system regeneration, a third set of experiments was performed to enhance the resolution of events (Table 1, set 3).

**Table 1:** Overview of regeneration experiments. Time shift between experiments was three days for sets 1 and 2, as well as eight hours for set 3. At each time point, given number of anterior and posterior regenerating specimens was fixed. The total number is a summation of all specimens fixed in the particular experiments. Please note, that due to the dissection, this number is half of the number of specimens fixed. Additional specimens listed in brackets were dissected in reserve and placed back in a stock aquarium after fixation of the last day or hour

Set	Experiment	Start date	Fixation [days after dissection]	Specimens fixed in each case	Total number of specimens
1	1a	13.08.2013	2, 6, 8, 14, 16, 20, 22, 28, 30	5 anterior/5 posterior	45 (+10)
	1b	16.08.2013	4, 10, 12, 18, 24, 26	5 anterior/5 posterior	30 (+10)
2	2a	21.08.2013	1, 5, 7, 13, 15, 19, 21	5 anterior/5 posterior	35 (+10)
	2b	24.08.2013	3, 9, 11, 17	5 anterior/5 posterior	20 (+10)
			Fixation [hours after dissection]		
3	3a	11.04.2014	52, 56, 60, 76, 80, 84	3 anterior/3 posterior	18 (+3)
	3b	11.04.2014	48, 64, 68, 72, 88, 92, 96	3 anterior/3 posterior	21 (+3)

### Immunohistochemistry

Anatomical details of body musculature and nervous system were investigated using standard immunohistochemical staining protocols and a range of well-established antisera. Standard negative controls were performed for all antisera, and in all cases the omission of the primary and/or secondary antiserum resulted in no staining. For analyses of the musculature, f-actin (=filamentous muscular actin) was stained with phalloidin-rhodamine and, for nervous system staining, antibodies against acetylated α-tubulin (structural component of microtubules, amongst others present in axons), FMRFamide (= small neuropeptide), and serotonin (=5-HT, neurotransmitter) were used.

Fixed whole mount animals were permeabilized in PBSTplus (0.1 mol l^−1^ PBS containing 0.1 % NaN_3_ and 2 % Triton X-100) for 1 h and blocked in PBST-NGS (0.1 mol l^−1^ PBS containing 0.1 % NaN_3_ and 0.1 % Triton X-100 with 6 % normal goat serum) overnight at RT. This step was followed by incubation in the primary antibody solution, either anti-FMRFamide (polyclonal antiserum raised in rabbit against fish and mammalian FMRFamide, supplied from Incstar, Stillwater, MN, USA, obtained via Acris Antibodies GmbH, Herford, Germany; dilution 1:500 in PBST-NGS), or a mixture of anti-acetylated α-tubulin (monoclonal anti-tubulin, acetylated antibody, produced in mouse, ascites fluid, Sigma-Aldrich, St. Louis, MO, USA; dilution 1:500 in PBST-NGS) and anti-serotonin (5-HT (Serotonin) rabbit antibody, lyophilized whole serum, Immunostar/Acris antibodies, Herford, Germany; dilution 1:500 in PBST-NGS) for 3 d at RT. After several rinses in PBST for about 4 h and PBST-NGS for 2 h, specimens were incubated in the secondary antibody solution, either only Alexa Fluor® 488 goat anti-rabbit IgG (H + L) (Invitrogen/Life Technologies, Darmstadt, Germany; dilution 1:500 in PBST-NGS) or a combination of this secondary antibody with Alexa Fluor® 568 goat anti-mouse IgG (H + L) (Invitrogen/Life Technologies, Darmstadt, Germany, dilution 1:500 in PBST-NGS) for 2 d at RT. Subsequently, specimens were rinsed in 0.1 mol l^−1^ PBS for at least 4.5 h (combined anti-acetylated α-tubulin/anti-serotonin staining), or 3 h (anti-FMRFamide staining) followed by incubation in a solution containing rhodamine-labelled phalloidin (Invitrogen, Carlsbad, CA, USA; 5 μl methanolic stock solution in 500 μl 0.1 mol l^−1^ PBS) overnight for additional anti-f-actin staining. Subsequently, specimens were dehydrated in an ascending isopropanol series, treated in Murray's clearing solution (benzyl alcohol plus benzyl benzoate, 1:2), and mounted in DPX (dibutyl phthalate xylene, Sigma-Aldrich, St. Louis, MO, USA) between to cover slips.

### Confocal microscopy and image processing

Specimens were analyzed with a confocal laser scanning microscope (Leica TCS STED, Leica Microsystems, Wetzlar, Germany). Confocal image stacks were processed with Leica LAS AF v2.3.5. and Leica LAS AF lite 3.3.10134 (both Leica Microsystems), as well as Fiji [19]. Drawings and final panels were designed using Adobe Photoshop CS6 and Illustrator CS6 (San Jose, CA, USA).

### Scanning electron microscopy (SEM)

Specimens for SEM were anaesthetized in 7 % MgCl_2_, fixed in Bouin’s solution (picric acid saturated aqueous solution, 37 % aqueous formaldehyde, and glacial acetic acid, 15:5:1) and dehydrated in an increasing ethanol series, ending with three changes of absolute ethanol. This was followed by critical point drying (CPG 030, BAL-TEC Union Ltd., LI) and sputtering with gold (Sputter Coater E5100 Series II 'Cool', Polaron Equipment Ltd., Watford, GB). SEM was performed using a Leitz 1000A (Wetzlar, DE). Drawings and final image plates were compiled as described above.

## Results

### Morphology of musculature and nervous system of adult specimens

The nervous system of adult specimens consists of a prominent anterior brain and a ventral nerve cord with distinct segmental nerves (Figs. 1a; 4a-b). The brain is situated in the prostomium and orientated towards anterior (Fig. 1a, b). While the brain always appears to be a compact structure on anti-serotonin and anti-acetylated-α-tubulin staining (Figs. 3j; 4a, b), anti-FMRFamide staining revealed a bipartite organization (Fig. 1a). Furthermore, in this staining there is a circular area without immunoreactivity in median posterior position (Fig. 1a, arrow). The brain is linked to the ventral nerve cord via two circumesophageal connectives (Fig. 1a). Each circumesophageal connective is composed of several neurite bundles, organized in a dorsal and a ventral root. Due to their fusion, an assignment of the particular neurite bundles to the roots is complicated in adult specimens, but possible based on their redevelopment during regeneration (see below). The ventral nerve cord is divided into two main strands, each of which is composed of several neurite bundles (Figs. 1a; 4a, b), but their exact number cannot be determined. One pair of ganglia can be found in each chaetiger, as well as in the peristomium. The ganglia of one segment almost contact at the midline of the body, thus making it impossible to elucidate the number of commissures connecting these ganglia (Fig. 3h, g). Three segmental nerves per ganglion run towards lateral. Whereas the first and third nerve is often stained weakly, the second segmental nerve, which extends into the parapodia, shows a more pronounced staining (Figs. 1a; 3 f, h; 4b).

The body wall musculature of *T.* cf. *punctata* is composed of an outer circular and an inner longitudinal layer (Figs. 1c-f; 4c-h). The circular musculature is most prominent at level with the chaetae, but present throughout the entire body (Figs. 1c, d, f; 4d-h). The longitudinal layer is composed of four main strands, two of them located dorsal and two ventral. The dorsal longitudinal muscle strands form a plate, encircling the dorsal half of the body in more anterior segments and up to two thirds in posterior segments, with a border directly above the notochaetae (Fig. 1e, f, i). The ventral longitudinal muscle strands are more compact, reaching to the notochaetae and are penetrated by the neurochaetae, thus appearing partially bipartite (Fig. 1c, e; i). On its course to the posterior end, the longitudinal body wall musculature gets weaker and fuses inside the pygidium (Fig. 1d). At the anterior end, the longitudinal muscle strands fan out in the prostomium (Fig. 1c, i). There is an additional longitudinal muscle fiber in ventro-median position (Figs. 1e; 4d, e), which runs over the whole length of the body. Another bundle of muscle fibers with dorso-ventral orientation is running through the posterior part of the brain (Fig. 1a, c, arrow). Further musculature can be found associated with the parapodia, comprising those of the aciculae and chaetae, as well as a meshwork-like one around the intestine (Fig. 1e, f).  Additionally, the mouth opening is supported by a large muscular pouch on its posterior side (Fig. 1c).

### Regeneration progress

**Fig. 2:**
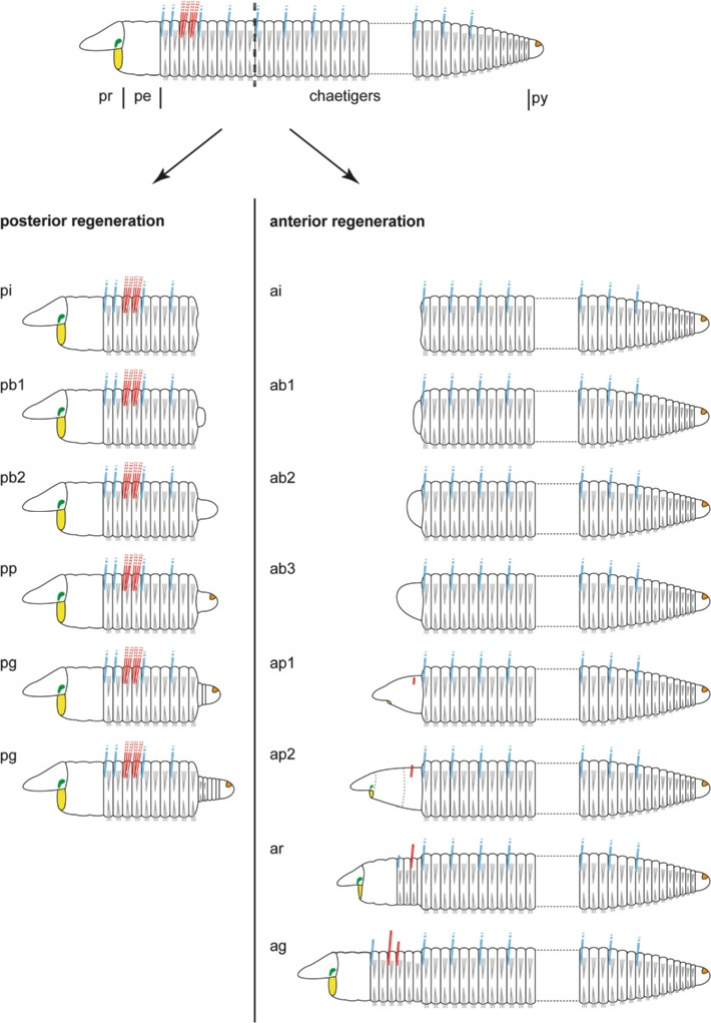
Schematic drawings of anterior and posterior regenerative stages in *Timarete* cf. *punctata*. The anus is colored in orange, branchiae in blue, chaetae in grey, mouth opening in yellow, nuchal organ in green, and tentacles in red. The dotted line in the upper drawing indicates the side of dissection. Please note that anterior and posterior stages show differences in characteristics and period. Posterior regeneration (left column) started with an invagination stage (pi, 0–2 days after disection), followed by a blastema stage (pb1-2, 3–5 dad) with formation and development of a blastema. Afterwards, during the blastema patterning stage (pp, 4–6 dad) the anus became visible. During the growth stage (pg, from 6 dad onwards) new segments were added by a posterior segment addition zone (SAZ) directly anterior to the pygidium (py). The first stages of anterior regeneration with invagination (ai, 0–1 dad) and blastema formation (ab1-3, 2–4 dad) are comparable to the posterior regeneration, according to the outer morphology. In the early blastema patterning stage (ap1, 4–5 dad) the mouth opening redeveloped. Afterwards, the first pair of tentacles occurred (6–8 dad) and the boundaries of pro- (pr) and peristomium (pe) as well as the nuchal organs became visible by the late blastema patterning stage (ap2, 6–10 dad). With the formation of three segments at once, the re-segmentation stage (ar, 6–11 dad) was reached and continued with sequential addition of three more segments. Also branchiae and a second pair of tentacles were seen first at this stage. Finally, during the growth stage (ag, from day 11 onwards), all described structure increased in size until they reached an adult condition

The survival rate of dissected specimens was up to 100 % in the different experimental settings. However, regeneration rates differed markedly between specimens. Moreover, specimens of the third set (Table 1) appeared to regenerate slower. To ensure the comparability of the presented data, we categorized regenerative stages (Fig. 2) according to external and internal morphological characteristics: Anterior regeneration is scaled in an invagination (ai), three blastema (ab1-3), two blastema patterning (ap1-2), a re-segmentation (ar), and a growth (ag) stage. Posterior regeneration comprises an invagination (pi), two blastema (pb1-2), a blastema patterning (pp) and a growth (pg) stage. The time specifications given below are approximated.

#### Anterior regeneration

**Fig. 3:**
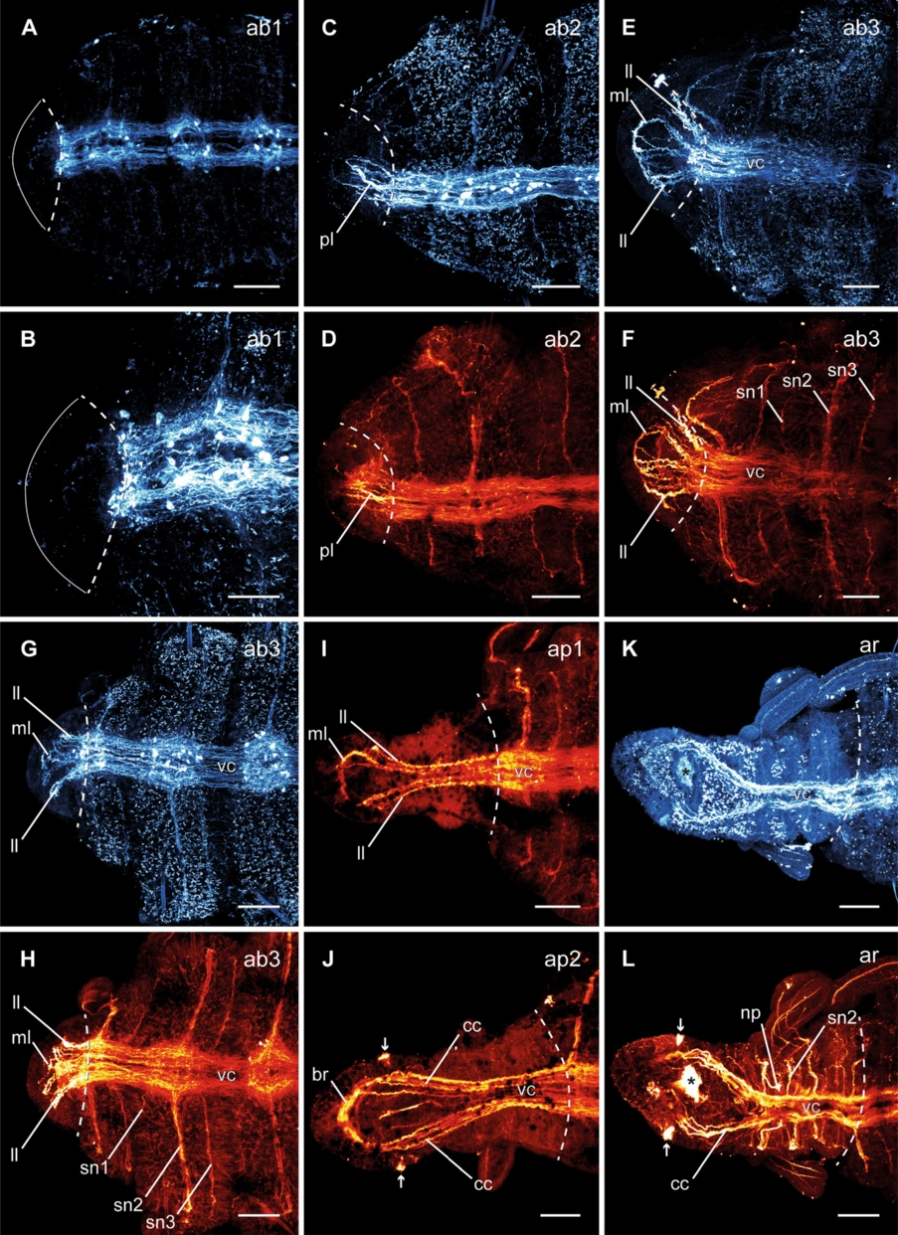
Different stages of anterior neuronal regeneration in *Timarete* cf. *punctata*. Anti-serotonin (cyan; A-C, E, G, K) and anti-acteylated α-tubulin (red; D, F, H-J, L) staining, confocal maximum projections. Anterior is left, all views are ventral showing the anterior end. The dotted white line indicates the side of dissection. Regeneration process is staged according to Fig. 2, time after decapitation of correspondent specimen is given in brackets. **a**, **b** Early blastema stage (ab1, 48 h). The nervous system has not been started to infiltrate the blastema (anterior margin indicated by white line). **c**, **d** Middle blastema stage (ab2, 68 h). Neurite bundles infiltrated the blastema, thereby forming a plexus (pl). **e**, **f** Late blastema stage (ab3, 92 h). The neurite bundles condensed into a three-loop-structure, composed of two lateral (ll) and one median loop (ml). The median loop (ml) is connecting the inner neurites of the ventral nerve cord (vc), whereas the lateral loops (ll) are solely connected to the outer neurites of their side. **g**, **h** Late blastema stage (ab3, 96 h). In this specimen the nerve loops (ll, ml) are more defined, but also bent more dorsal. **i** Early blastema patterning stage (ap1, 96 h). According to the elongation of the blastema, the nervous system was stretched anterior. Simultaneously, the transition of the nerve loops to the roots of the later circumesophageal connectives occured. While the median loop (ml) simply elongated, the lateral loops (ll) converged to one structure. **j** Late blastema patterning stage (ap2, 7 dad). The transition of the nerve loops to the circumesophageal connectives (cc) had finished. At the anterior end, the brain (br) had redeveloped. Also the nuchal organs (arrows) were visible, now. **k**, **l** Re-segmentation stage (ar, 11dad). Redevelopment of segmental nerves (sn2) according to reestablished segments was visible. Scale bars = 50 μm, except of A = 25 μm

**Fig. 4:**
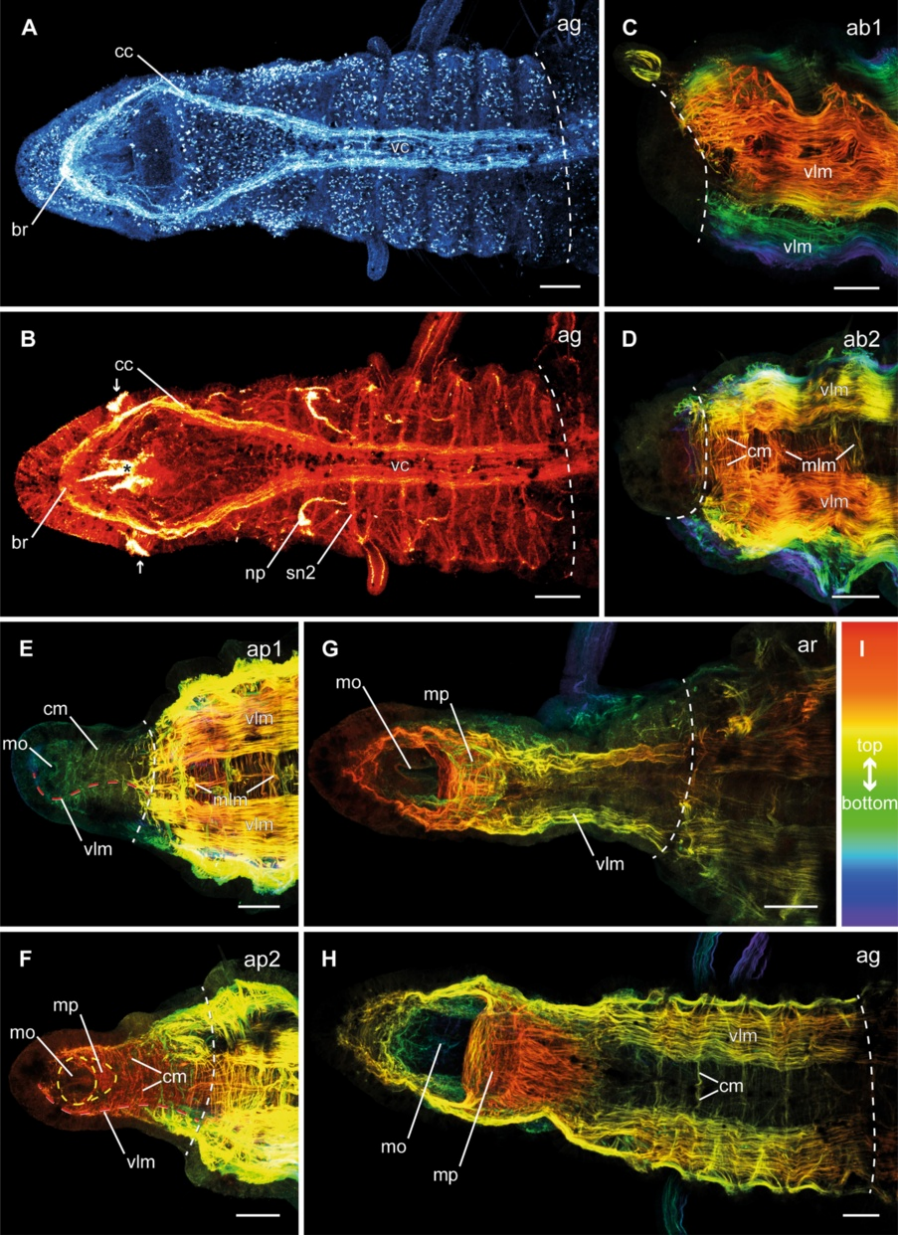
Different stages of anterior neuronal and muscular regeneration in *Timarete* cf. *punctata*. Anti-serotonin (cyan; A), anti-acetylated α-tublin (red; B), and anti-f-actine (depth coded, legend in I; C-H) staining, confocal maximum projections. Anterior is left, all views are ventral showing the anterior end, except C which is a latero-ventral view. The dotted white line indicates side of dissection. Regeneration process is staged according to Fig. 2, time after decapitation of correspondent specimen is given in brackets. **a**, **b** Growth stage (ag, 14 d). The nervous system almost reached its final shape. Aside from the size and the hardly definable segmental ganglia, the brain (br), the circumesophageal connectives (cc), as well as the ventral nerve cord (vc) with its segmental nerves (sn2) were present. **c**, **d** Early blastema stage (ab1, 48 h) and middle blastema stage (ab2, 72 h). The musculature still remained bluntly cutted without infiltration inside the blastema. **e** Early blastema patterning stage (ap1, 96 h). Thin circular (cm) and longitudinal muscle fibers (vlm) were visible inside the elongated blastema. The ventral longitudinal muscle fibers (vlm) were already organized in two strands, having an hourglass-like course (red dotted line). Further musculature started surrounding the mouth opening (mo). **f** Late blastema patterning stage (ap2, 5 d). The muscular elements became more prominent and the two strands of the ventral longitudinal musculature (vlm) diverged from each other to their final shape (dotted red line). The musculature of the mouth opening (mo) formed a pouch (mp) at its posterior edge. **g** Re-segmentation stage (ar, 9 d). The muscular pouch of the mouth opening (mp) continued its development and the ventral longitudinal musculature (vlm) now reached its final shape. **h** Growth stage (ag, 14 d). All muscular elements possessed an almost adult shape, now. Scale bars = 50 μm

Following dissection, the musculature contracted to close the wound, and thus caused an invagination at the cutting side (Fig. 2, ai). One day after dissection (dad), all investigated specimens remained in this stage. By the second day, first indications of a developing blastema (Fig. 2, ab1) became visible. The third and fourth day after dissection were characterized by elongation of the blastema (Fig. 2, ab2–3). This was followed by a redevelopment of the mouth opening at 4–5 dad (Fig. 2, ap1) and the occurrence of the first pair of tentacles by 6–8 dad (Fig. 2, ap1–2). In all investigated specimens this pair redeveloped at the position of the later chaetiger three. During the late blastema patterning stage, also the boundary of prostomium and peristomium became slightly visible. The re-segmentation started with the first three segments at once by 6–11 dad (Fig. 2, ar). Afterwards, three more segments redeveloped sequentially (Figs. 3k, l; 4a, b). The maximum number of anteriorly regenerated segments was six and was redeveloped earliest at 11 dad. The redevelopment of further tentacles and the redevelopment of branchiae did not appear to cluster around any specific time point and, especially for the branchiae, no obvious pattern was visible. A second pair of tentacles occurred first in a specimen of 9 dad, this time at chaetiger 4. First branchiae were found at 7 dad. When all six segments were established, they grew to their final size (Fig. 2, ag). After 30 dad, most new anterior ends were nearly of same size, color and pigmentation compared to the residual segments. Nevertheless, they had fewer tentacles and branchiae than undissected specimens.

#### Posterior regeneration

Invagination (0–2 dad) and blastema formation (3–5 dad) were comparable to anterior regeneration (Fig. 2, pi and pb1–2). The first signs of blastema patterning was the formation of the anus at 4–6 dad (Fig. 2, pp). Afterwards, new segments were added successively always directly in front of the pygidium, so that the oldest newly developed segments were in the anteriormost position (Fig. 2, pg). About one week after dissection, the number of new segments was five to eight, and after two weeks up to 22. The first branchiae were observed at 20 dad in a specimen with 25 new segments. Likewise to anterior regeneration, there was no fixed pattern of branchiae occurrence recognizable. Finally, during the fourth week after dissection, the transition from the first ten (residual) to the new developed (regenerated) segments became increasingly indistinct and specimens were no longer distinguishable from untreated ones.

### Anterior neuronal and muscular regeneration

In the early blastema stage, no neuronal regeneration was detectable (Fig. 3a, b). With growth of the blastema, an infiltration of neurites into the blastema occurred. Within less than 3 dad, a plexus had developed during the middle blastema stage (Fig. 3c, d). These neurites originated in the ventral nerve cord of the residual body. Also first signs of organization by fusion of neurites to bundles occurred at this stage, at least as visible in the anti-serotonin staining. By the end of 4 dad, a state exhibiting several neuronal loops was reached within the late blastema stage (Fig. 3e–h). These loops included one median and two lateral loops. The neurites of the median loop were connected with the inner neurites of each strand of the ventral nerve cord (Fig. 3e, f), whereas each lateral loop was connected exclusively with the outer ipsilateral neurites of the ventral nerve cord. Depending on the specimen, the loops were orientated more anterior (Fig. 3e, f) or more dorsal (Fig. 3g, h), and showed different levels of mergence. During the end of the first week after dissection, the loops were stretched anterior according to blastema elongation and started to fuse (Fig. 3i). At the end of blastema patterning, the nerve loops were no longer distinguishable, as they were completely fused to the circumesophageal connectives (Fig. 3j). An assignment of the neurites respectively to the dorsal and ventral roots of the circumesophageal connective was impossible in some cases, but the outer ones which in particular showed a more intense staining referred to the dorsal root, whereas the inner ones represented the ventral root. Furthermore, the brain had redeveloped at the most anterior end of the regenerated nervous system and the nuchal organs became visible due to their ciliation. During the second week after dissection, the reformation of segments was accompanied by the redevelopment of the segmental nerves (Fig. 3k, l). As in adult specimens, the second segmental nerve was already the most prominent one. On reaching the growth stage, the regeneration of the nervous system in the redeveloped anterior body end was complete (Fig. 4a, b).

The first signs of redeveloping body wall musculature occurred in the early blastema patterning stage in the middle of the first week after dissection (Fig. 4c–e). At this time point, thin longitudinal muscle fibers with an hourglass-like shape, first circular muscle fibers with origin in the lateral blastema, and a thin muscle ring surrounding the mouth opening became visible (Fig. 4e). Once started, the redevelopment of the musculature continued and during the late blastema patterning stage the circular layer became prominent (Fig. 4f). Furthermore, the musculature of the mouth opening started to redevelop the muscular pouch. With the early re-segmentation in the second week after dissection the longitudinal musculature was well redeveloped and the ventral longitudinal muscle strands departed from each other to form their final shape (Fig. 4g). The development of the muscular pouch of the mouth opening also continued. When all regenerated segments were present in the growth stage by two weeks after dissection, all components of the body wall musculature as well as those of the mouth opening possessed their final shape (Fig. 4h).

### Posterior neuronal and muscular regeneration

**Fig. 5:**
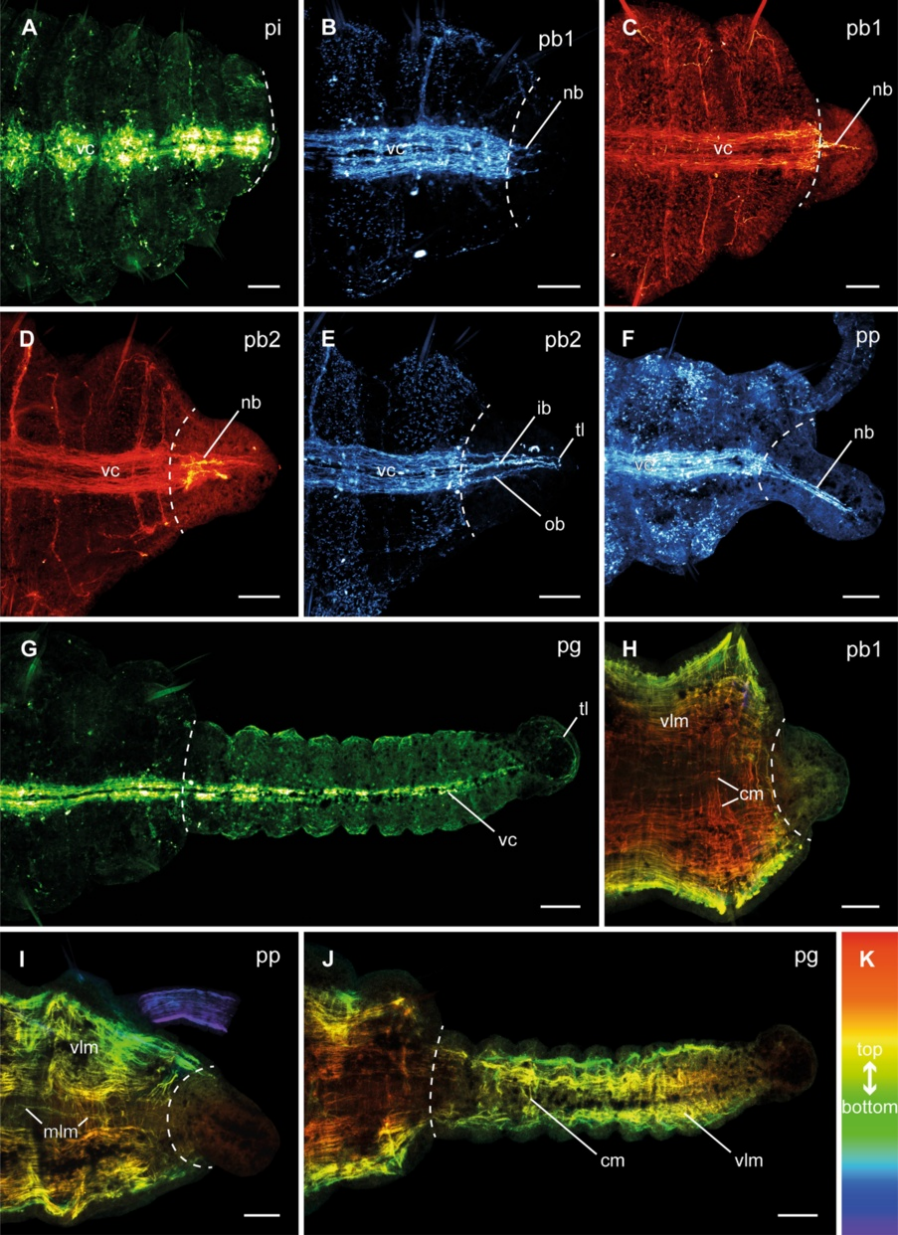
Different stages of posterior neuronal and muscular regeneration in *Timarete* cf. *punctata*. Anti-FMRFamide (green, A, G), anti-serotonin (cyan; B, E, F), and anti-acetylated α-tublin (red; C, D), as well as anti-f-actine (depth coded, legend in K; H-J) staining, confocal maximum projections. Anterior is left, all views are ventral showing the posterior end. The dotted white line indicates side of dissection. Regeneration process is staged according to Fig. 2, time after decapitation of correspondent specimen is given in brackets. **a** Invagination stage (pi, 48 h). **b** Early blastema stage (pb1, 60 h). Neurite bundles (nb), originated in the residual ventral nerve cord (vc) infiltrated the blastema. In this specimen, the outer neurite bundles grew faster than the inner ones. **c** Early blastema stage (pb1, 96 h). In this specimen, the inner neurite bundles grew faster than the outer ones. **d**, **e** Late blastema stage (pb2, 92 h). The neurite bundles have grown out. While in anti-acetylated α-tublin staining only a plexus of neurite bundels (nb) was visible, the anti-serotonin staining revealed inner (ib) and outer (ob) neurite bundles. The inner ones formed a loop (tl) in the most posterior position. **f** Blastema patterning stage (pp, 96 h). The neurite bundles (nb) represented a more compact structure, now. **g** Growth stage (pg, 12 d). The ventral nerve cord was well developed in the older regenerated segments, but faded the more posterior it run. **h** Early blastema stage (pb1, 96 h). Inside the blastema no muscular elements were detectable. **i** Blastema patterning stage (pp, 96 h). Also at this time point, there was no visible regeneration of musculature. **j** Growth stage (pg, 12 d). The older the segments were, the more developed the musculature was. In the younger segments, especially the circular musculature (cm) was rarely present. Scale bars = 50 μm

Posterior nervous system regeneration started with an ingrowth of neurite bundles into the blastema in the early blastema stage (Fig. 5a–c) at the third day after dissection. While in most specimens the first neurite bundles originated in the inner neurites of the ventral nerve cord (Fig. 5c), in some specimens these originated in the outer ones (Fig. 5b). During the late blastema stage at four days after dissection the neurite bundles had grown and originated in all parts of the residual ventral nerve cord (Fig. 5d, e). In comparison with the anti-acetylated α-tubulin staining (Fig. 5d), the anti-serotonin staining (Fig. 5e) visualized more compact bundles. Furthermore, they were clearly assignable to the inner and outer parts of the ventral nerve cord. The inner neurite bundles connected at the most posterior position, thus forming a terminal loop. When regeneration continued, the neurite bundles fused and formed the final ventral nerve cord (Fig. 5f). With the addition of subsequent segments, the posterior nervous system showed its adult shape (Fig. 5g).

The redevelopment of the body wall musculature is described only briefly, as in most cases the staining did not allow the resolution of all details. During the blastema stages as well as the blastema patterning stage, no muscle fibers were detectable inside the regenerating posterior body part (Fig. 5h, i). The longitudinal muscle strands and circular fibers first became visible in redeveloped segments (Fig. 5j). In older regenerated segments, both layers were well defined, but the younger the segments the more indistinct the musculature was, especially in the circular one. In the youngest regenerated segments no muscular elements were detectable.

## Discussion

### Adult morphology

The basic architecture of body wall musculature in *Timarete* cf. *punctata* is comparable to that in other cirratulids [20, 21]. Compared with *T. anchylochaeta* [21] (named as *Audouinia anchylochaeta* Schmarda, 1861; source of synonymy: [22, 23]), the ventral longitudinal muscle strands are of comparable shape and extent in the anterior body. Differences occur in the dorsal longitudinal muscle strands, which cover only one third of the body in *T. anchylochaeta*. In comparison with *Cirratulus* cf. *cirratus* [20], there are some differences in the shape of longitudinal muscle strands; the dorsal longitudinal musculature of *C.* cf. *cirratus* is completely fused to a plate and the ventral longitudinal muscle strands are more compact. Future studies are necessary to understand if the differences between these cirratulids are reflected by the phylogeny.

Longitudinal muscle layers composed of four main strands have been identified in several annelid families and this feature is thought to represent a part of the hypothetical myoanatomical ground pattern [24–27]. A ventral longitudinal muscle fiber also exists in other annelid families [28–31]. As has been shown in numerous investigations, a well-developed circular musculature is typical for burrowing annelids [26], a lifestyle also typical for our investigated species. The muscular pouch of the mouth opening is part of the ventral pharyngeal organ, a structure already described in cirratulids [20, 32].

A dorso-ventral muscle bundle penetrating the brain of *T.* cf. *punctata* is not detectable in *C.* cf. *cirratus* [20]. However, distinct muscle fibers with similar orientation are described for *Ctenodrilus serratus* [33]. In this species, these fibers divide the brain in an equally sized anterior and posterior neuropil. Contrary, in *T.* cf. *punctata* the penetration of muscle fibers lies in between the posterior commissures or between the brain and a nerve directly behind it, which cannot be finally clarified. However, muscles extending in a dorso-ventral direction through the prostomium are also described in other Cirratuliformia, such as *Cossura pygodactylata* [28].

Apart from this, the nervous system of *T.* cf. *punctata* is largely comparable to that of *C.* cf. *cirratus* [20], as expected due to the close phylogenetic relationship (Additional file 2: Figure S1). Both possess a tetraneuralian ventral nerve cord with three segmental nerves leaving each ganglion laterally.

The brain is orientated anteriorly and the circumesophageal connectives are composed of two roots each. However, in adult specimens of *T.* cf. *punctata* the dorsal and ventral roots of the circumesophageal connective as well as the paramedian and main connectives of the ventral nerve cord are fused, so that distinguishing between these is only possible based on their redevelopment during regeneration. This underlines the importance of developmental studies to investigate nervous system architecture, as already suggested by Müller [34]. Based on her hypothesis, annelids possess a pentaneuralian ventral nerve cord in the ground pattern. Comparing our findings with the phylogeny, Cirratulidae are part of the Cirratuliformia, which comprise amongst others also the Flabelligeridae, and are the sister taxon of the Siboglinidae [10, 35]. Together with the Orbiniida they constitute a sister clade to the other Sedentaria [35]. Neither in the flabelligerid *Poeobius meseres* [36] nor in the siboglinid *Lamellibrachia satsuma* [37] the median connective is described, but it is present in the orbiniids *Proscoloplos cygnochaetus* and larvae of *Scoloplos armiger* [38, 39]. Thus, the absence of a median connective in the ventral nerve cord might represent an autapomorphy of the Cirratuliformia/Siboglinidae clade. The existence of three segmental nerves represents the typical condition in Cirratulidae and reflects the supposed ancestral state in annelids [40, 41]. Typically, the nerve that innervates the parapodial appendages is the thickest [39]. Although parapodial cirri are absent in cirratulids, the most prominent segmental nerve is also the second one, which runs to the parapodia, so that the innervations of further parapodial structures can be assumed.

### Regeneration process

Cirratulids are known for their extensive regeneration capability [20, 42, 43], which is also frequently used during asexual reproduction [12, 15]. Compared with the detailed descriptions of anterior regeneration in *Cirrineris* sp. [42] and *Cirratulus* cf. *cirratus* [20], *Timarete* cf. *punctata* shows an average speed of regeneration. While in the latter the maximum number (see below) of regenerated anterior segments is first reached after 11 days, it takes eight in *Cirrineris* sp., but 14 in *C.* cf. *cirratus*. Interestingly, a blastema is visible in *T.* cf. *punctata* on the second day after dissection, but in *Cirrineris* sp. the first signs of a blastema are visible not earlier than by the third day. This suggests that anterior regeneration is initiated earlier in *T.* cf. *punctata*, but needs longer for full redevelopment of all structures. Furthermore, the tentacles were redeveloped in *C.* cf. *cirratus* and *Cirrineris* sp. at the same time with the start of re-segmentation, but develop prior to re-segmentation in *T.* cf. *punctata*. All three cirratulids show a nearly constant number of regenerated anterior segments, which is always less than removed. It appears that they have a kind of minimal functional unit composed of prostomium, peristomium and a limited number of chaetigers (six to seven in *Cirrineris* sp., five in *C.* cf. *cirratus*, six in *T.* cf. *punctata*) which is exclusively regenerated. This observation is in line with investigations in different annelid taxa [43–45]. However, in others the number of anterior regenerated segments usually depends on the cutting side [46–48].

Notably, the sequence in which the anterior segments were regenerated differs between the investigated cirratulids: While *C.* cf. *cirratus* always regenerates all anterior segments at once, *Cirrineris* sp. first regenerates four and *T. cf. punctata* only three segments and the remaining ones were regenerated subsequently [20, 42]. This might imply the existence of an anterior growth zone as supposed for several syllid species [29, 49, 50]. Furthermore, this contradicts the conclusion of Balavoine [3], that sequential addition of segments is absent during annelid anterior regeneration. Given that *T.* cf. *punctata* starts with three segments, there is a striking similarity to the ontogeny, where the larvae develops the first three chaetigers at once and all others are generated subsequently by the (posterior) segment addition zone between the last chaetiger and the pygidium [9]. Transferring this to *T.* cf. *punctata*, a reactivation of a larval developmental program might explain the findings. However, there is no support for such a reinitiation of larval developmental patterns during regeneration in *Cirrineris* sp. and *C*. cf. *cirratus* [20, 42]. An alternative explanation is that the remaining segments are already determined but poorly developed.

Another interesting point is the order of tentacle redevelopment: Based on the occurrence of tentacles, cirratulid genera can be subdivided in three groups: (1) without tentacles (former Ctenodrilidae), (2) one pair of tentacles (bitentaculate; e.g., *Aphelochaeta*, *Chaetozone*, *Dodecaceria*), or (3) two or more groups of tentacle filaments (multitentaculate; e.g., *Cirratulus*, *Cirriformia*, *Timarete*) [51, 52]. The absence of tentacles was shown to be a derived character e.g., [53], but it is unknown, if the bitentatuclate or the multitentaculate genera represent the ancestral stage. Based on our findings, we hypothesize that the occurrence of more than one pair of tentacles is the derived character: In *T.* cf. *punctata*, one pair is regenerated first on chaetiger three (later groups one and two) and the second one then follows on chaetiger four (later groups three and four). This implies that this first pair on chaetiger four represents a duplication of tentacles, otherwise simultaneous development may be accepted. Subsequently, the number of tentacles increases in all four groups. Interestingly, in other multitentaculate cirratulids the branchiae are developed first [54, 55] or together with the first tentacles [56] during ontogeny.

During posterior regeneration, the pygidium and the posterior segment addition zone (SAZ) are redeveloped. This is followed by segment addition, which is comparable to normal growth, as supposed by Balavoine [3] or Gazave et al. [57]. Given that this process of posterior regeneration is widespread in annelids [29, 50, 58, 59], it presumably represents a plesimorphic condition. Nevertheless, Moment [60] reported a nearly simultaneous formation of all regenerated segments in the maldanids *Clymenella torquata* and *Axiothella mucosa*, while *A. rubrocincta* again showed serial segment addition. This exception may be due to the constant number of segments present in most maldanids [61].

Finally, it is clear that regeneration speed varies between specimens within one set of experiments as well as between different sets. Because experimental parameters were kept constant, these differences should be based on individual characters: *T.* cf. *punctata* showed frequent architomy, thus specimens of same size could have very different ages and consequentially different regeneration speed. Also nutritional conditions may be important. Differences between the first two sets and the third one could be based on the time shift and the potentially different situation of individuals within their respective reproductive cycle (e.g., change between growth and asexual reproduction) which can slow down regeneration due to limited resources [62–64].

### Regeneration of musculature and nervous system

The regeneration of the body wall musculature during posterior regeneration mirrors the normal posterior growth [9, 65, 66]. During anterior regeneration, there are differences in the redevelopment of the circular and the longitudinal layer: While longitudinal musculature shows an outgrowth originated in its correspondent structures within the residual (old) body, the circular musculature develops independently inside the blastema. The same was found in other annelid species as well, which also show a slower regeneration of the musculature as compared to the nervous system [20, 67].

**Fig. 6:**
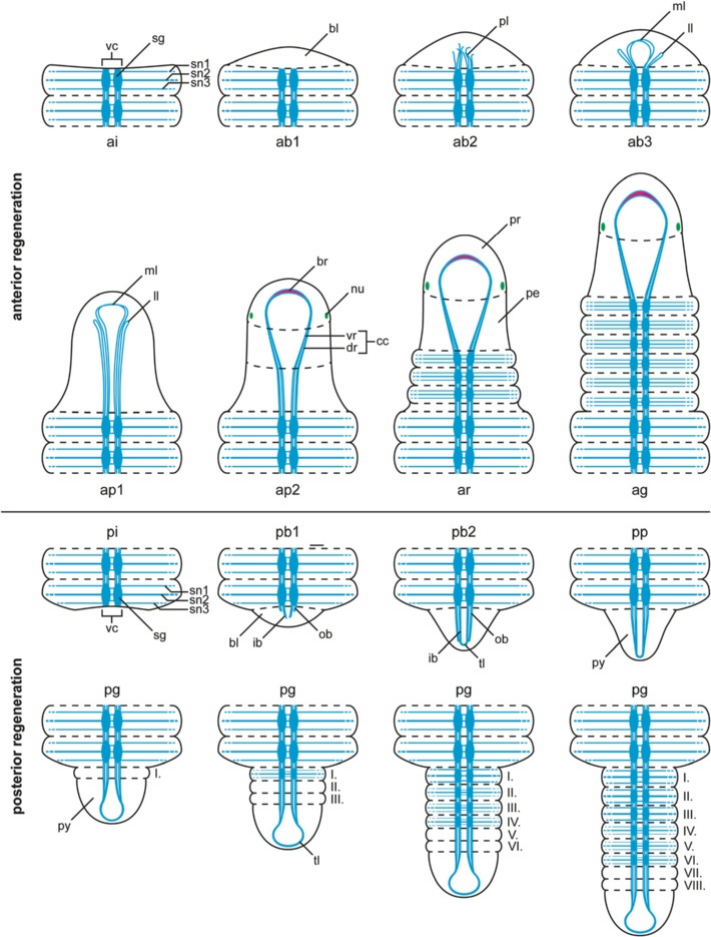
Schematic overview of nervous system redevelopment during anterior and posterior regeneration in *Timarete* cf. *punctata*. Regenera darw1ti darwon process is staged according to Fig. 2. The brain is colored in pink, the residual nervous system in light blue and the nuchal organs in green. Roman numbers in posterior regeneration refer to regeneration order. Anterior regeneration stages: ai, invagination stage; ab1-3 blastema stages; ap1-2 blastema patterning stages; ar, re-segmentation stage; ag, growth stage. Posterior regeneration stages: pi, invagination stage; pb1-2, blastema stages; pp blastema patterning stage; pg, growth stage. Further abbreviations: bl, blastema; br, brain; cc, circumesophageal connective; dr, dorsal root of cc; ib, inner neurite bundle; ll, lateral nerve loop; ml, median nerve loop; nu, nuchal organ; ob, outer neurite bundle; pe, peristomium; pl, plexus; pr, prostomium; py, pygidium; sg, segmental ganglion; sn1-3, segmental nerves 1–3; tl, terminal loop; vc, ventral nerve cord; vr, ventral root of cc

During anterior regeneration the first signs of the redeveloping nervous system are outgrowing nerve fibers with origin in the ventral nerve cord of the residual body (Fig. 6), which is comparable to other annelids [67–70]. This is followed by the development of a three-loop structure, as in *Cirratulus* cf. *cirratus* [20]. However, there are differences in *Timarete* cf. *punctata*. First, the median loop is orientated anteriorly from the outset and, in addition, it is initiated in a compressed position. Later, this structure is stretched according to blastema elongation. Afterwards, all three loops fuse to form the circumesophageal connectives, in which the median loop becomes the ventral root and the lateral loops the dorsal root of the circumesophageal connectives. The occurrence of three closed loops is not described outside of cirratulids, but in *Dorvillea bermudensis* a related structure composed of a closed median loop connected to the inner neurites of the ventral nerve cord and two lateral neurite bundles connected each to the outer parts of the ventral nerve cord is reported [70]. Moreover, circumscribable roots of the circumesophageal connectives were also found in amphinomids, enchytraeids, naidids, or spionids [69, 71, 72]. Segmental nerves were redeveloped together with their segments, but ganglia are hardly visible at this stage. However, they must be present to interconnect the segmental nerves with the ventral nerve cord.

The nervous system redevelopment during posterior regeneration shares some major similarities with the anterior one. There is also an outgrowth originated in the residual ventral nerve cord and a loop-like structure occurs as well (Fig. 6). However, this structure solely refers to the inner neurite bundles. Later, the outer ones also fuse with this terminal loop. This terminal loop is presumably homolog to the terminal commissure found in *D. bermudensis* [70]. The main difference between anterior and posterior nervous system regeneration is the time point of reoccurrence for segmental nerves and ganglia: During anterior regeneration they redevelop together with the segments, while in posterior regeneration their redevelopment is delayed until the new segments have reached a certain age.

## Conclusions

In this study we investigated the redevelopment of external structures, the musculature, and the nervous system during posttraumatic regeneration. Although early anterior and posterior regeneration with wound healing, blastema formation and patterning are largely comparable, both processes show remarkable differences in later redevelopment. While posterior regeneration appears to be a recapitulation of "normal" growth, anterior regeneration shows a unique pattern with similarities to the ontogeny of segment formation. Remarkably, the redevelopment of the nervous system during anterior and posterior regeneration is realized with loops connecting both main strands of the ventral nerve cord, which were later stretched. Given that related processes were found in other annelids, too, this might represent a plesiomorphic condition.

Furthermore, we demonstrate how regeneration studies can yield new insights into anatomical patterns, ontogeny, and evolutionary processes. Although well investigated in some model annelids, such as *Capitella teleta* or *Platynereis dumerilii*, data for most annelid taxa are lacking. In these annelids, especially in those for which breeding is impossible, regeneration studies represent a powerful tool to close the gaps in our knowledge.

## Additional files

Additional files 1:
**Supplementary information.** Additional material and methods for Supplementary Figure S1. Additional files 2: Figure S1. Maximum likelihood tree based on 16S and CO1 sequences of available *Timarete* and *Cirriformia* sequences.

## Abbreviations

ab1-3: Blastema stage 1–3 of anterior regeneration
ag: Growth stage of anterior regeneration
an: Anus
ai: Invagination stage of anterior regeneration
ap1-2: Blastema patterning stages 1–2 of anterior regeneration
ar: Re-segmenation stage of anterior regeneration
be: Branchiae
bl: Blastema
br: Brain
cc: Circumesophageal connective
cm: Circular musculature
dad: Days after dissection
dlm: Dorsal longitudinal musculature
dr: Dorsal root of circumesophageal connective
in: Intestine
ib: Inner neurite bundle
ll: Lateral nerve loop
ml: Median nerve loop
mlm: Median ventral longitudinal muscle fiber
mo: Mouth opening
mp: Muscular pouch of mouth opening
nb: Neurite bundle
np: Nephridium
nu: Nuchal organ
ob: Outer neurite bundle
pb1-2: Blastema stage1-2 of posterior regeneration
pe: Peristomium
pg: Growth stage of posterior regeneration
pl: Plexus
pi: Invagination stage of posterior regeneration
pm: Musculature associated with the parapodia
pp: Blastema patterning stage of posterior regeneration
pr: Prostomium
py: Pygidium
sg: Segmental ganglion
sn1-3: Segmental nerves 1–3
tl: Terminal loop
vc: Ventral nerve cord
vlm: Ventral longitudinal musculature
vr: Ventral root of circumesophageal connective
