# Supplementary material for: Morphological investigations of posttraumatic regeneration in Timarete cf. punctata (Annelida: Cirratulidae)
Source: Zoological Lett. 2015 Aug 6;1:20. doi: 10.1186/s40851-015-0023-2 (PMC4657251; doi:10.1186/s40851-015-0023-2)
Supplement: Additional files 1: — Supplementary information. Additional material and methods for Supplementary Figure S1. [file 40851_2015_23_MOESM1_ESM.docx]

**Supplementary information to:**

**Morphological investigations of posttraumatic regeneration in *Timarete* cf. *punctata* (Annelida: Cirratulidae)**

Weidhase M, Helm C, Bleidorn C

**Material and methods**

We obtained all available 16S and CO1 sequences of the genera *Timarete* and *Cirriformia* as well as the outgroup sequences from the NCBI GenBank (Supplementary table 1). The 16S and CO1 sequences were separately aligned with MAFFT v7 [1] and masked for phylogenetic analysis with Gblocks Server v0.91b [2]. Subsequently, both alignments were concatenated by using FASconCAT v1.0 [3] and maximum likelihood analysis was conducted using RaxML v7.0.3 [4] choosing the GTR + Γ + I-model and 1,000 bootstrap replicates. The phylogenetic tree was visualized with FigTree v1.4.0 (Andrew Rambaut) and Adobe (San Jose, CA, USA) Illustrator CS6. Pairwise sequence distances were calculated with MEGA v6.06 [5] using the K2P-model.

**References**

1. Katoh K, Standley DM. MAFFT Multiple Sequence Alignment Software Version 7: Improvements in Performance and Usability. Mol Biol Evol. 2013;30(4):772-80.

2. Castresana J. Gblocks Server. Barcelona: Castresana Lab, Institut de Biologia Evolutiva; 2002.

3. Kück P, Meusemann K. FASconCAT. Bonn: Zool. Forschungsmuseum A. Koenig; 2010.

4. Stamatakis A. RAxML-VI-HPC: maximum likelihood-based phylogenetic analyses with thousands of taxa and mixed models. Bioinformatics. 2006;22(21):2688-90.

5. Tamura K, Stecher G, Peterson D, Filipski A, Kumar S. MEGA6: Molecular evolutionary genetics analysis version 6.0. Mol Biol Evol. 2013;30(12):2725-9.

6. Magalhães WF, Seixas VC, Paiva PC, Elias R. The multitentaculate Cirratulidae of the genera *Cirriformia* and *Timarete* (Annelida: Polychaeta) from shallow waters of Brazil. PLOS One. 2014;9(11):e112727.

**Supplementary figure 1**

Maximum likelihood tree based on 16S and CO1 sequences of available *Timarete* and *Cirriformia* sequences. Bootstrap values are given for relevant branches. Numbers in brackets refer to supplementary table 1. Specimens of *T.* cf. *punctata* show K2P distances in comparison with the Brazilian specimens of 2.7-2.9 % for 16S respectively 5.6 % for CO1. K2P distances within Brazilian specimens are 0-0.2 % for 16S and 0 % for CO1 [6]. Based on this, *T.* cf. *punctata* used in this study represents presumably a distinct, maybe cryptic, species. Please note that the genera *Cirriformia* and *Timarete* appear to be polyphyletic but together constitute a monophyletic group.

**Supplementary table 1**

Overview of 16S and CO1 sequences used for phylogenetic reconstruction including their origin.

|  | **Species** | **Origin** | **16S** | **CO1** |
| --- | --- | --- | --- | --- |
| 01 | *Timarete* cf. *punctata* | Aquarium, University of Leipzig, Germany | KT033731 | KP794936 |
| 02 | *Timarete punctata* | São Pedro e São Paulo Archipelago, Brazil | KM192199 | KM192182 |
| 03 | *Timarete punctata* | Salvador, Brazil | KM192203 | KM192186 |
| 04 | *Timarete punctata* | São Pedro e São Paulo Archipelago, Brazil | KM192198 | KM192181 |
| 05 | *Timarete punctata* | Salvador, Brazil | KM192204 | KM192187 |
| 06 | *Timarete punctata* | Rocas Atoll, Brazil | KM192202 | KM192185 |
| 07 | *Timarete punctata* | São Pedro e São Paulo Archipelago, Brazil | KM192197 | KM192180 |
| 08 | *Timarete punctata* | Salvador, Brazil | KM192205 | KM192188 |
| 09 | *Timarete punctata* | Rocas Atoll, Brazil | KM192201 | KM192184 |
| 10 | *Timarete punctata* | Rocas Atoll, Brazil | KM192200 | KM192183 |
| 11 | *Cirriformia chicoi* | Rio de Janeiro, Brazil | KM192189 | KM192164 |
| 12 | *Cirriformia chicoi* | Rio de Janeiro, Brazil | - | KM192165 |
| 13 | *Timarete caribous* | São Pedro e São Paulo Archipelago, Brazil | - | KM192170 |
| 14 | *Timarete caribous* | São Pedro e São Paulo Archipelago, Brazil | KM192193 | KM192171 |
| 15 | *Timarete caribous* | Fernando de Noronha, Brazil | KM192190 | - |
| 16 | *Timarete caribous* | Rocas Atoll | - | KM192174 |
| 17 | *Timarete caribous* | Fernando de Noronha, Brazil | - | KM192168 |
| 18 | *Timarete caribous* | Fernando de Noronha, Brazil | KM192191 | KM192166 |
| 19 | *Timarete caribous* | Fernando de Noronha, Brazil | KM192192 | KM192167 |
| 20 | *Timarete caribous* | Rocas Atoll | - | KM192173 |
| 21 | *Timarete caribous* | Abrolhos Archipelago | - | KM192177 |
| 22 | *Timarete caribous* | Abrolhos Archipelago | - | KM192175 |
| 23 | *Timarete caribous* | Abrolhos Archipelago | - | KM192176 |
| 24 | *Timarete caribous* | Rocas Atoll | - | KM192172 |
| 25 | *Timarete ceciliae* | Fernando de Noronha, Brazil | KM192196 | - |
| 26 | *Timarete ceciliae* | Salvador, Brazil | KM192195 | - |
| 27 | *Timarete ceciliae* | Salvador, Brazil | KM192194 | KM192178 |
| 28 | *Timarete ceciliae* | Fernando de Noronha, Brazil | - | KM192178 |
| 29 | *Cirriformia capixabensis* | Espirito Santo, Brazil | - | KM192161 |
| 30 | *Cirriformia capixabensis* | Espirito Santo, Brazil | - | KM192162 |
| 31 | *Cirriformia capixabensis* | Espirito Santo, Brazil | - | KM192163 |
| 32 | *Cirriformia tentaculata* | Saint-Efflam, Brittany, France | KT033725 | KP794930 |
| 33 | *Cirratulus* cf. *cirratus* | Aquarium, University of Leipzig, Germany | KT033724 | KM083601 |
| 34 | *Dodecaceria concharum* | Helgoland, Germany | KT033729 | KP794934 |
