# Supplementary figures and images for: Morphological investigations of posttraumatic regeneration in Timarete cf. punctata (Annelida: Cirratulidae)
Source: Zoological Lett. 2015 Aug 6;1:20. doi: 10.1186/s40851-015-0023-2 (PMC4657251; doi:10.1186/s40851-015-0023-2)

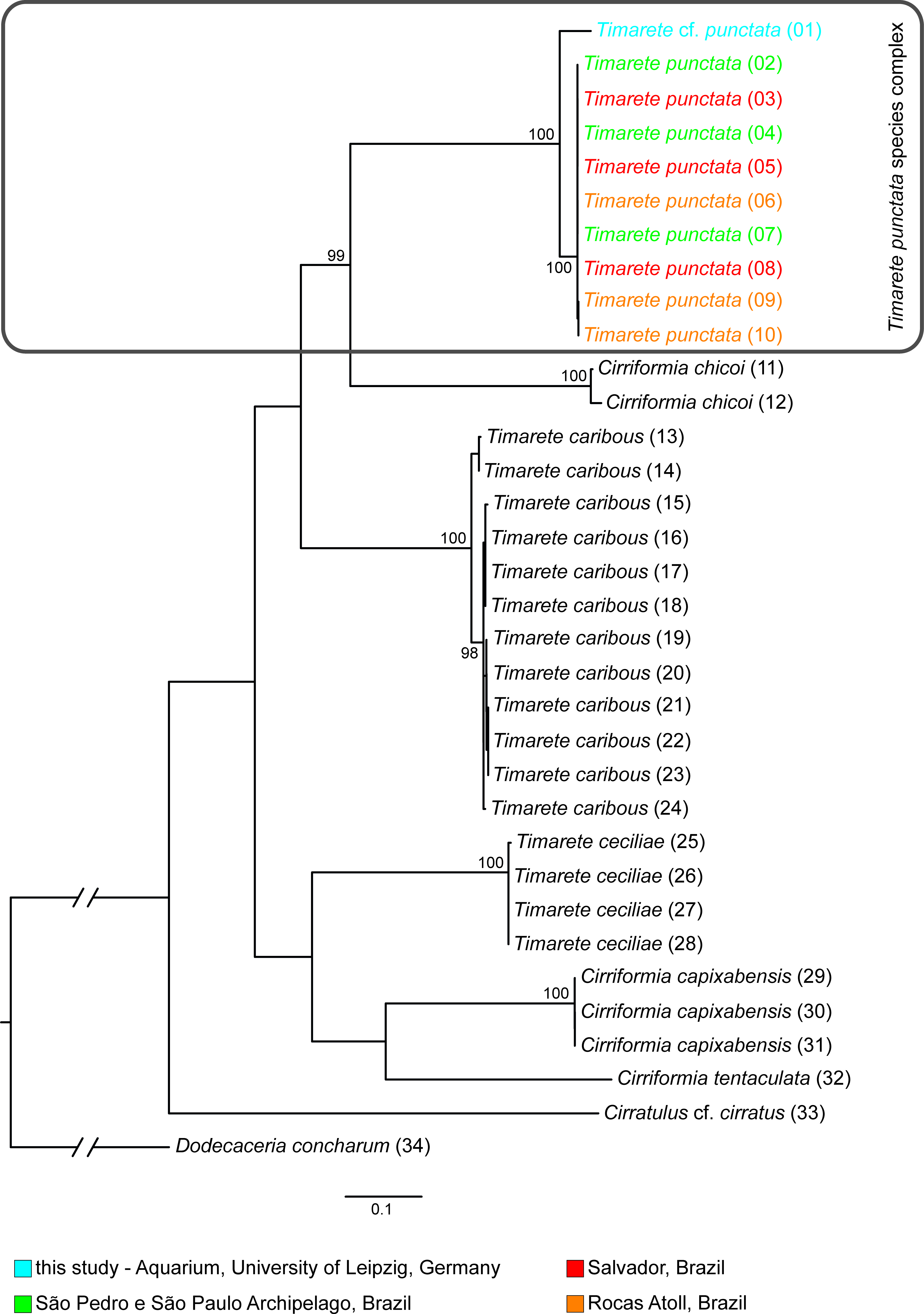

Supplement: Additional files 2: Figure S1. — Maximum likelihood tree based on 16S and CO1 sequences of available Timarete and Cirriformia sequences. [file 40851_2015_23_MOESM2_ESM.tif]
